# Low-cost prototyping of nitinol wires/frames using polymeric cores and sacrificial fixtures with application in individualized frames anchoring through the atrial septum

**DOI:** 10.1038/s41598-023-48106-4

**Published:** 2023-12-09

**Authors:** Hemanta Dulal, Trey Swan, Subhi J. Al’Aref, Seyedhamidreza Alaie

**Affiliations:** 1 Department of Mechanical and Aerospace Engineering, New Mexico State University, Las Cruces, NM USA; 2 Division of Cardiovascular Medicine, Department of Internal Medicine , University of Arkansas for Medical Sciences, Little Rock, AR USA

**Keywords:** Biomedical engineering, Mechanical engineering

## Abstract

Self-expanding frames for minimally invasive implants are typically made from nitinol wires and are heat treated to maintain the desired shapes. In the process of heat treatment, nitinol structures are placed in a high-temperature oven, while they are confined by a fixture. During this process, nitinol exerts a high amount of force. Accordingly, a fixture requires high mechanical strength and temperature resistance; this is why fixtures are typically made from metals. The use of metal fixture also increases the turnaround time and cost. However, accelerating this process is beneficial in many applications, such as rapid development of medical implants that are patient-specific. Inspired by the use of sacrificial layers in microfabrication technology, here we propose a novel method for shape setting nitinol wires using a sacrificial metal fixture. In this process, the nitinol wires are first aligned inside copper hypotubes. Next, the forming process is done using hand-held tools to shape complex geometrical structures, annealing the nitinol reinforced by copper, and then selectively etching copper hypotubes in ammonium persulfate solutions. In this process, other sacrificial cores, which are 3D printed or cast from low-cost polymers, are also used. This combination of polymeric cores and minimal use of metals enables reducing the cost and the turnaround time. As a proof of concept, we showed that this process was capable of fabricating springs with mm or sub-mm diameters. The result showed a change of less than 5% in the intended diameter of the nitinol spring with diameters ranging from ~ 0.7 to 1.9 mm, which confirms copper as a suitable sacrificial fixture to obtain the desired complex geometry for nitinol. A metric, based on the elastic strain stored in copper is suggested to predict the possible variation of the intended dimensions in this process. Finally, to demonstrate the potential of this method, as proof of concept, we fabricated NiTi wire frames designed for anchoring through the atrial septum. These frames demonstrated septal defect occluders that were designed based on a patient’s cardiac image available in the public domain. This low-cost rapid fabrication technique is highly beneficial for a variety of applications in engineering and medicine with specific applications in rapid prototyping of medical implants.

## Introduction

Shape setting nitinol (NiTi) wires^1–3^ is among the methods used for fabrication of self-expanding frames used in minimally invasive implants. In the process of shape setting, nitinol structures are traditionally placed in an oven at ~ 500 °C^4,5^, while they are confined by a fixture^3^. During this process, nitinol undergoes a high amount of stress, which requires a fixture with high mechanical strength at the specified high temperature. This is why fixtures are typically made from metals^4^. However, this need poses both a challenge and high cost for rapid prototyping and testing of nitinol structures due to the requirement for fabricating metal fixtures. Direct 3D printing or machining metal fixtures are expensive processes. Alternatively, the use of a reconfigurable fixture, made from pins and plates^6^, simplifies the process of fabricating fixtures, however, it has limited capability in forming complex geometries. More recently, ceramic^7,8^ and wooden^9^ fixtures with local Joule’s heating effect were introduced; however, they still have limitations on forming more complex geometries. Therefore, cost-effective annealing of nitinol are still under investigation^5,10, 11^.

Use of sacrificial layers or structures is a concept that is widely used in microfabrication^12–14^ especially the lift-off process^15^, however, in this context the layers are used as a spacer rather than load-bearing structures. This process involves choosing a sacrificial layer on which the main material will be deposited. Subsequently, the sacrificial material and etchants/solvent^16^, are chosen such that the sacrificial material can be selectively etched/dissolved without damaging the main structure. Copper (Cu) as a sacrificial material, in the lift-off process, has been used for fabrication of nitinol microelectromechanical systems (MEMS)^17^, and micropatterned thin films^18,19^. The latter two reports^18,19^ investigated the use of Cu as a sacrificial layer for fabrication of thin and thick film patterned NiTi. Such patterned NiTi films were also suggested^19^ to be used as an additional layer in covering stents. Another example of this lift-off process is fabrication of thin film NiTi thermostat arrays deposited on sacrificial Cu and chromium (Cr) layers^20^. Despite these reports on the utility of Cu as sacrificial material in deposition of free standing NiTi films, the use of Cu as sacrificial fixtures for annealing NiTi has been less explored, in the literature, to our best knowledge. This use of Cu as a sacrificial layer in the lift-off process suggests the possibility of its use as a sacrificial fixture (load-bearing components) for the shape setting, which is the focus of this work.

The advantages of a reliable and cost-effective process for rapidly iterating nitinol frames (in the forms of sheets, wires, or tubes) are significant for the research and development of minimally invasive implants and devices. Many implants or medical devices rely on self-expanding frames that are fabricated from various forms of wires, sheets, tubes, etc. Few examples include, implantable sensors (CardioMEMS™)^21^, left atrial appendage occluders (WATCHMAN™)^22^, stone extractors (NCircle^®^)^23^, stent grafts^24^, and transcatheter aortic valves^25^. In addition, recently there has been an interest in methodologies for designing and fabricating patient-specific implants^26,27^, rather than conventional universal implants. A unique capability for this paradigm is the use of a patient’s medical imaging data for the design and fabrication of patient-specific implants. While this engineering approach is under investigations, the capability of this paradigm can be improved by development of processes that allow for rapid iterations of designing, fabricating, and testing an implant within a timeframe of few days. Although, several investigations have shown the feasibility of this paradigm for polymeric implants^27,28^, from an engineering perspective, limited success has been shown for nitinol implants. This need for exploring NiTi implants also motivates the current work to introduce a fabrication process to be employed for the rapid prototyping of transcatheter implants that leverage nitinol frames.

Here we propose adapting the concept from the lift-off process in microfabrication (use of sacrificial copper) for shape setting nitinol structures with a small form-factor. In this process, the ideal sacrificial fixture must be: (1) low cost, (2) easily deformable at room temperature, (3) able to tolerate stresses during the annealing process and, (4) could be etched selectively without damaging the annealed nitinol. In this work, we assess this possibility by shaping nitinol into typical shapes such as springs. We also assess the possibility to shape set nitinol with complex shapes with small and large form factors. We hypothesize that copper hypotubes, as the sacrificial layer, and ammonium persulfate as etchant, could be employed, given prior use of this chemical for selective etching of sacrificial copper^16^. We chose copper because (1) it enables large plastic deformation at room temperature^29^ (2) its melting temperature is well above nitinol’s annealing temperature, and (3) miniaturized hypotubes are commercially available. Although we were unable to find reports on the selective (NiTi) etch rate of nitinol in ammonium persulfate, it could be inferred from other work that nickel^16^ had insignificant etch rates in commercial solutions of ammonium persulfate (APS 100). In this work, we assess whether the nitinol structures with various shapes could be released completely. We also assess, if any detrimental surface chemistry or significant change in diameter is observed after releasing the structures.

Here, this work demonstrates the novel technique of copper reinforcement for the heat treatment of nitinol. This effort focuses on the processes that have applications in the rapid prototyping of self-expanding nitinol frames. Such frames have applications in the fabrication of medical implants, however, other applications in the aerospace industry (such as the mesh tires^30,31^) are also envisioned. Implantable NiTi frames are typically processed starting with NiTi tubes (e.g., stents^32^), plates (e.g., patterned NiTi sheets rolled and welded to form stents^2^), and wires (e.g., wire based stents^2,33^ and heart valves^2,33^). A review on self-expanding NiTi stents made from wires, sheets and tubes can be found elsewhere^2^. Although the use of NiTi tubes is the more common form in fabricating self-expanding implants/frames, other forms such as wire (Cragg Stent^2,33^, Boston Scientific-Symphony Stent^34^, and heart valve with crimped wires ^2,33^) or rolled patterned sheets^2^ have been also employed in commercial implants or research on NiTi stents. However, in this study, we limit the focus of this work to wire/frames with a small form factor. The success of this method can introduce new routes for development of the implants that require nitinol wire frames. Few examples are crimped NiTi frames^2,33^ used in heart valves, nitinol wire/frames in septal defect occluders^35^ or NiTi wire/frames in graft stents^36^ that are formed by cylinder and pin fixtures. The direct application of this technique is in any processes that involved shape setting NiTi wires, that are commonly done using fixtures and/or pins, and crimping/welding them into self-expanding framse^2,33, 34^.

We evaluate the introduced technique to explore its potentials. First, we assess it is capable of producing standard structures such as miniaturized springs with a reasonable variation of dimensions. Next, to demonstrate the concept, we employ it for the fabrication of self-expanding frames that are individualized for anchoring through the atrial septum based on physiological topologies derived from publicly available patients’ CT/MRI images^37,38^. The technique was shown to be able to produce complex topologies for frames designed to anchor on septum, which makes it suitable for rapid prototyping of individualized or highly customized self-expanding frames for implants such as atrial defect occluders. While these demonstrations of the concept are carried out by bending/winding wires with hand-held tools suitable for research laboratories, this technique can also be integrated with desktop/commercial computerized wire bending^39^ for industrial applications. Finally, our effort addresses the limitations of this process in producing topologies that originated, from a mechanics of materials viewpoint. This discussion elucidates routes for predicting the expected tolerance or improving it in various designs.

## Methods

**Figure 1:**
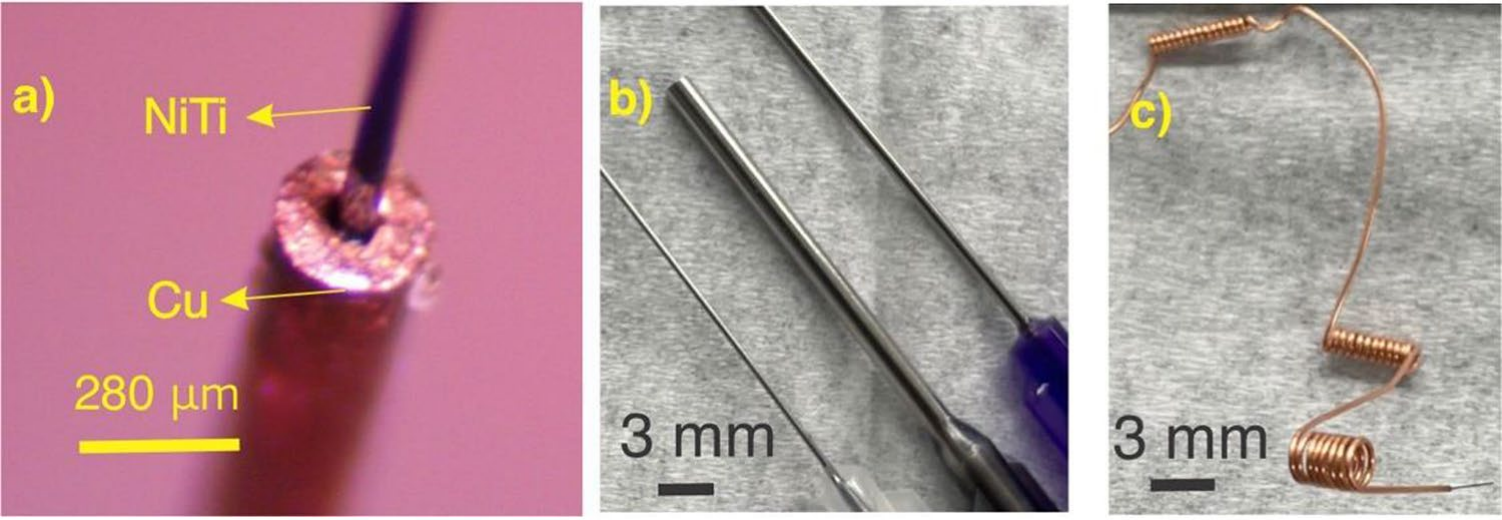
NiTi reinforced by copper hypotubes such that (**a**) copper protects nitinol (~ 70 $\mathrm{\mu m}$ diameter), (**b**) could be winded around a mandrel, and could **c)** form structures such as coils.

Superelastic nitinol wires were aligned inside copper hypotubes, and the wires were formed into the desired shapes (Fig. 1a). Nitinol wires with diameters of ~ 70 $\upmu$m (MALIN CO., nominal 0.003″) and ~ 150 $\upmu$m (Tegra Medical, nominal 0.006″) were used. The first was aligned in copper hypotubes with the nominal outer diameter of 300 $\upmu$m (Fig. 1a) and the latter was aligned inside copper tubes with a nominal outer diameter of 1 mm. The alignment was carried out manually with the assistance of a stereoscope. The hypotubes were sourced from an electrical discharge machining supplier. Forming various topologies was carried out through (1) forming by jewelry pliers, (2) winding around steel hypotubes (Fig. 1b), removing the mandrel (Fig. 1c), and (3) winding around a polymeric core (3D printed in Fig. 3f, or cast in Fig. 3d) and decomposing/dissolving/cutting the core. Since the selective etchant was not designed for metals other than NiTi and Cu, the mandrel/core was dislodged, or cut/dissolved if the core is polymeric. For more complex topologies (e.g., a septal defect occluder’s frame, Fig. 3d,f) the core was cut and then removed. This elimination of a core in the annealing process is the novelty of this work.

**Figure 2:**
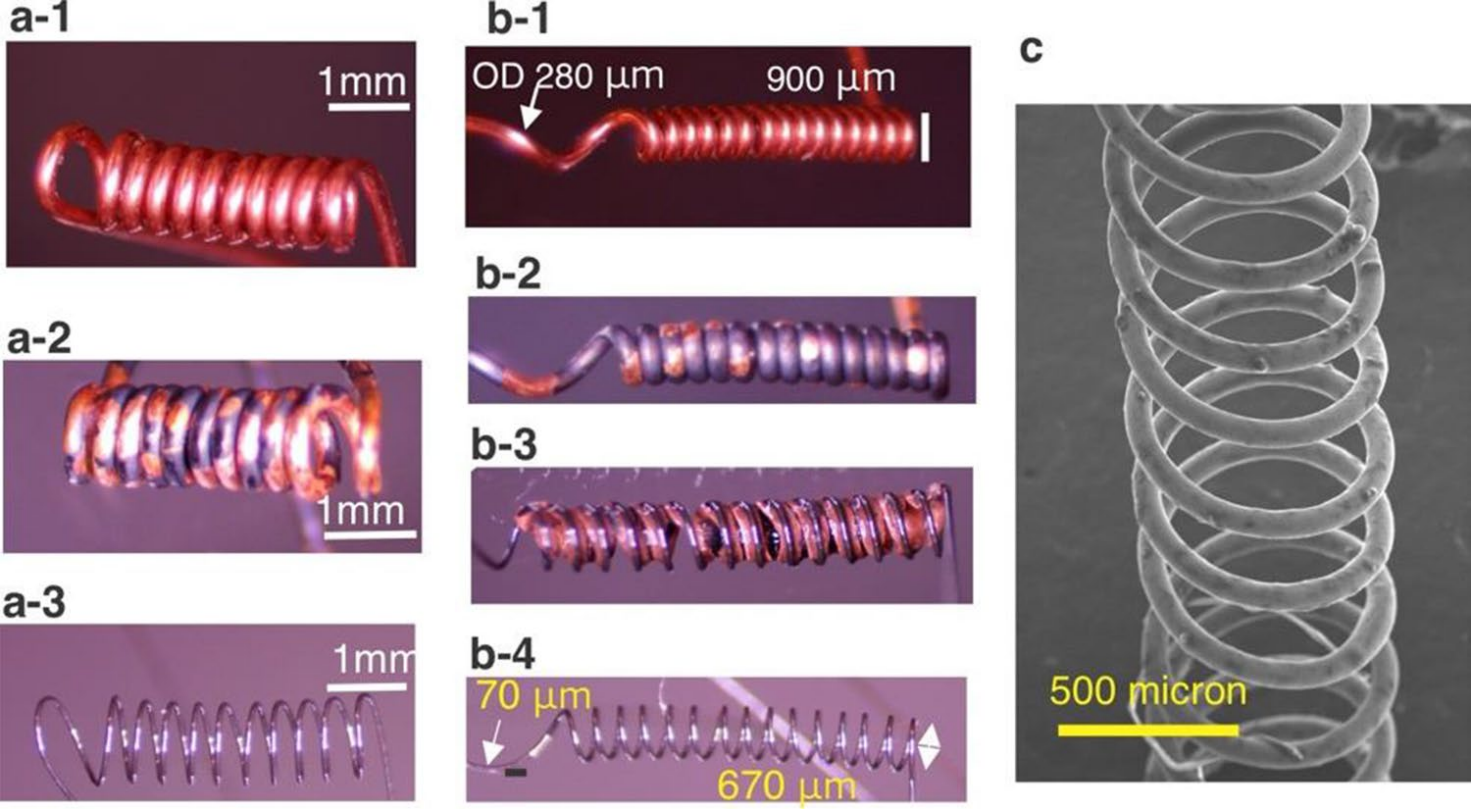
Miniaturized NiTi springs fabricated copper hypotubes yielding nitinol springs with diameters 1.1 mm in (**a-1**) a coil of copper/NiTi structure, (**a-2**) heat treated, and (**a-3**) released by etching. Another spring with diameter 0.75 mm is formed in (**b-1**) a coil of copper/NiTi that is (**b-2**) heat treated, (**b-3**) is gradually etched in ammonium persulfate solutions, and is (**b-4**) fully released. (**c**) SEM image of another spring with OD ~ 0.7 mm.

**Figure 3:**
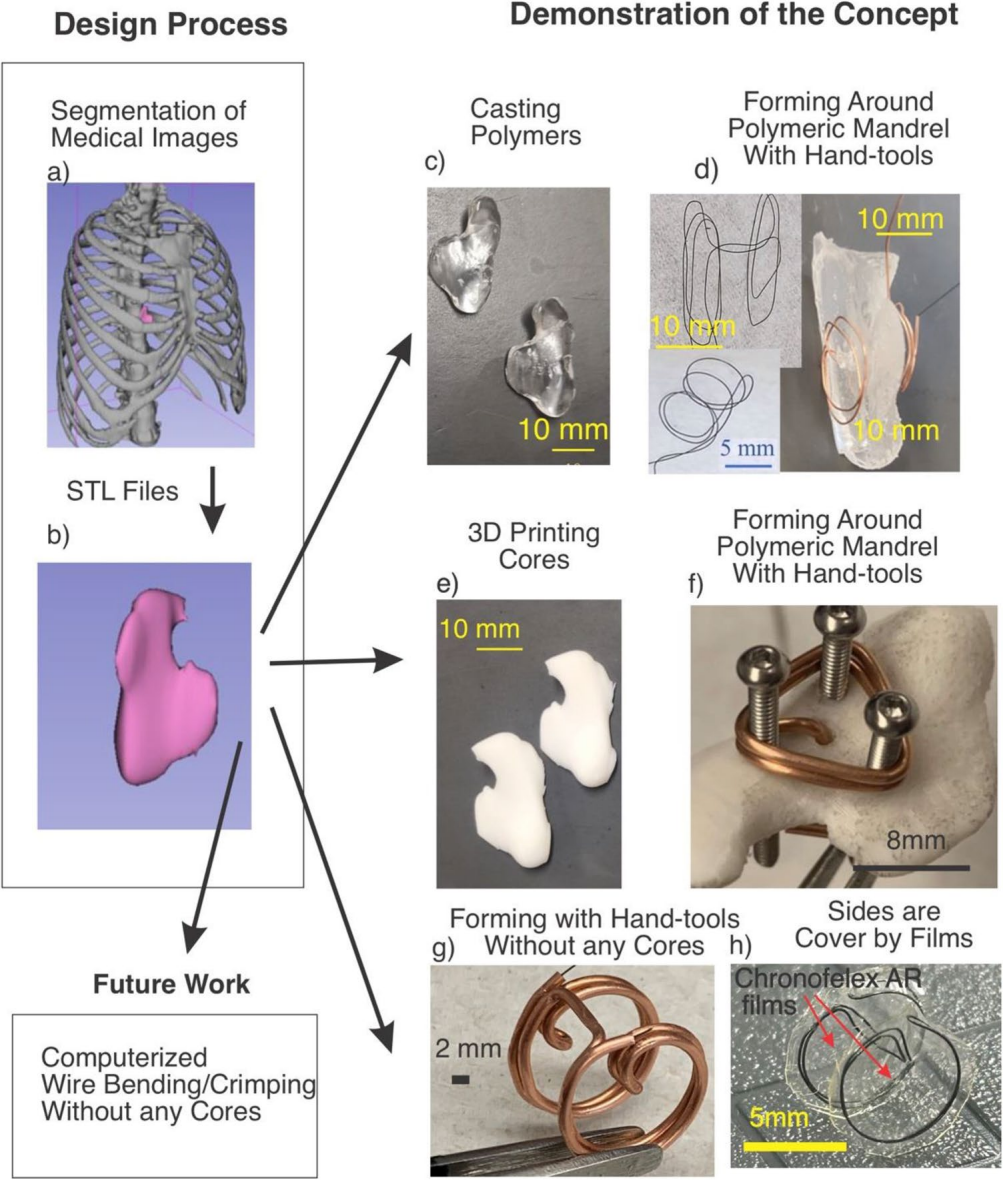
The demonstrations of the concept in this work: (**a**, **b**) inter-atrial septum (pink structure) are segmented. (**c**) PDMS cores are cast using 3D printed molds that follow the septum topology, (**d**) NiTi /Cu wires are wrapped that follow the topology on the PDMS cores, which can be heat treated and released (inset-top); another frame was fabricated by including tension during winding. (inset-bottom), **e)** plastic parts are directly 3D printed, (**f**) NiTi/Cu is wrapped with complex topologies, (**g**) complex topologies can be formed using typical wire bending using hand tools and without any cores, (**h**) NiTi frames are fabricated with the shape of an atrial defect occluder covered with hemocompatible films. The demonstrations suggest that the proposed technique can be integrated with a computerized wire bending that is ideal for a rapid prototyping platform.

**Figure 4:**
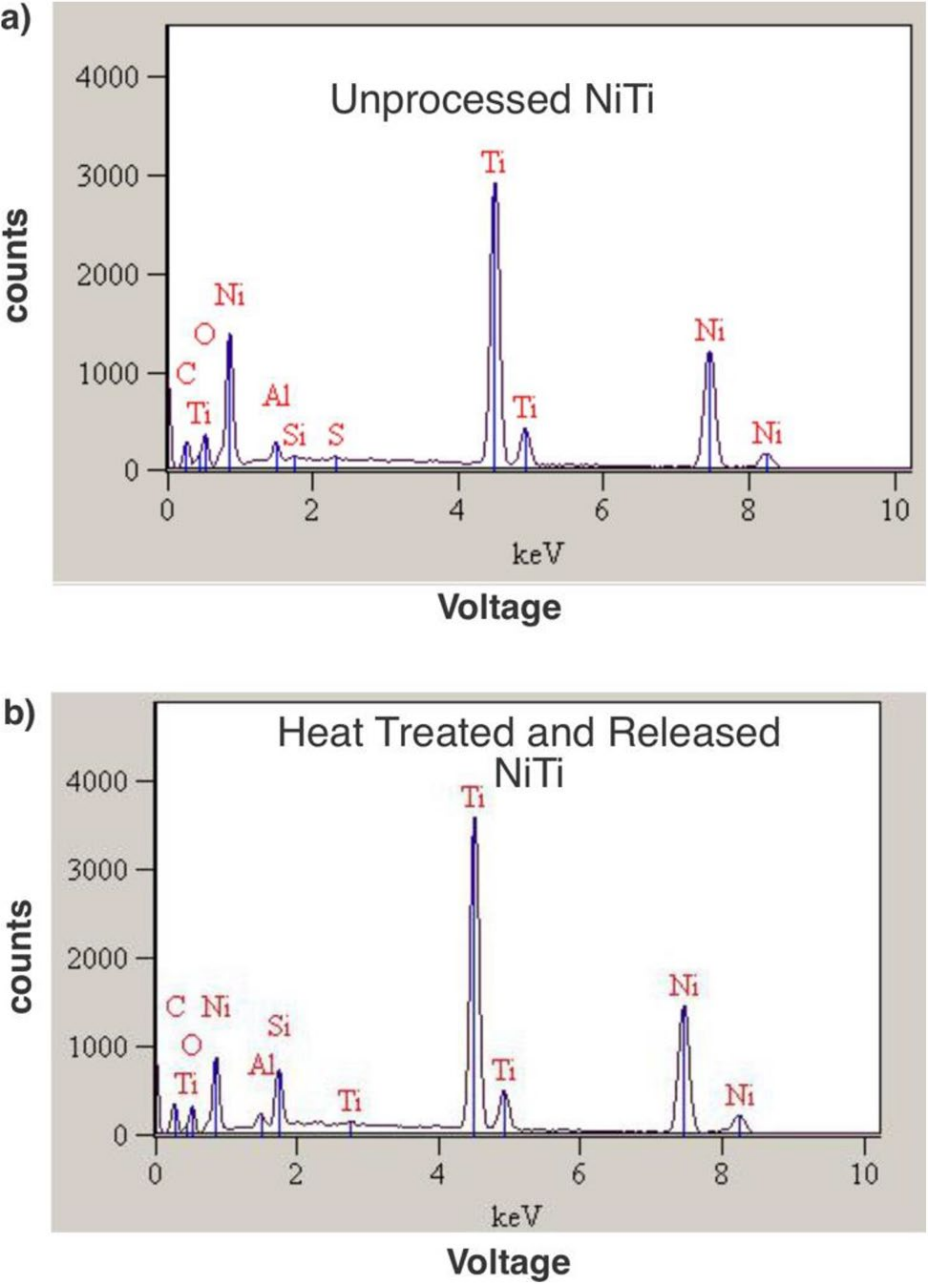
EDS analysis results for: (**a**) The surface of an untreated nitinol wire and (**b**) A nitinol wire after going through the proposed process. Al, Si, and C signatures could be attributed to the ambient dust/residues since they are not present in the chemical processing (e.g., etching copper). The analysis did not show any trace of copper.

Heat treatment and etching were carried out using a tube furnace in air^6^. The selective etchant for copper was ammonium persulfate. Its powder was dissolved in water with the weight ratio of 23%, and was subsequently used for etching copper (Fig. 2a,b). The furnace was heated to 500 °C and the temperature was monitored using a K-type thermocouple. The temperature variation inside the furnace was monitored to be ~ 25 °C. Each sample was heat treated for 3 min and quenched in water afterward. Subsequently, the dimensions of the samples were characterized using a stereoscope (Fig. 2a-3,b-4). The sample was left in the etchant for at least 6 h and the process was repeated with fresh etchant, if needed. After a complete release of samples, they were cleaned in water, dried using a nitrogen gun, and characterized using the stereoscope.

While annealing NiTi in air^6,8^ is a common practice, surface oxidation during the shape setting can form TiO_2_ and Ni-rich layers^40^. It is noted that an oxide layer is known to improve biocompatibility and/or engineering life of NiTi implants^41^. However, if necessary, the oxide layer can be optimized^42^, or any undesirable phases on the NiTi surface can be eliminated by passivation techniques, or chemical etching^43^. Other approaches that avoid oxidation are annealing in inert gases^6^ and vacuum^6^. In our setting, we expect the thickness of the oxide layer, to be less than 30 nm^40^. The use of salt bath^6^ can also improve the uniformity in the heat-treatment. While in this work we heat treated NiTi in air, adapting the ideal process parameters for heat or surface treatments (e.g., annealing in vacuum, or etching NiTi) is beyond the scope of this work. Nonetheless, we note that the usage of copper as a sacrificial fixture, in principle, could be integrated with any of the aforementioned annealing or surface treatments that can be studied in the future.

To assess whether NiTi is etched in ammonium persulfate solutions, we characterized a straight NiTi wire (0.003″ diameter) before and after the proposed processes using both an optical microscope and a micrometer. To estimate the lower bound of the elastic strain limits of the NiTi wires (0.003″ diameter), four wires were wrapped around cylinders and then released. At each step, we optically characterized whether they could recover their straight form after release. Gradually, the bending radius is reduced until the wire can no longer fully return to its initial form. Subsequently using beam theories, the corresponding elastic strain limit is estimated.

To assess the variation of dimensions in the heat-treated nitinol, nitinol springs were winded on steel needles. After releasing the springs, their spacing and diameters were characterized using the microscope. To demonstrate the utility of this technique, we also fabricated NiTi frames suitable for a septal defect occluder that is individualized for an anonymized patient in the public domain. For this purpose, we started with chest CT images^37^ and segmented out the atrial septum using the open-source Slicer 3D^38^ (Fig. 3a,b). The segmentation of the ribcage was carried out by defining the threshold color (Fig. 3a), whereas segmentation of the atrial septum (Fig. 3a,b) was done manually using the paint and draw command. Subsequently, the segmented septum was exported as .stl file format (Fig. 3b) and was 3D printed (Fig. 3e) using a low-cost Fused Deposition Modeling (FDM) printer (Anycubics Kobra). Next, silicone (Ecoflex™-30) molds were cast using the 3d printed septum, they were in turn used to cast the septum made from polydimethylsiloxane (PDMS, Sylgard^®^ 184) which is flexible but firmer than the silicone (Fig. 3c,d). Alternatively, the septum could also be 3D printed from Polyvinyl alcohol (PVA) filaments, which are water-soluble.

**Figure 5:**
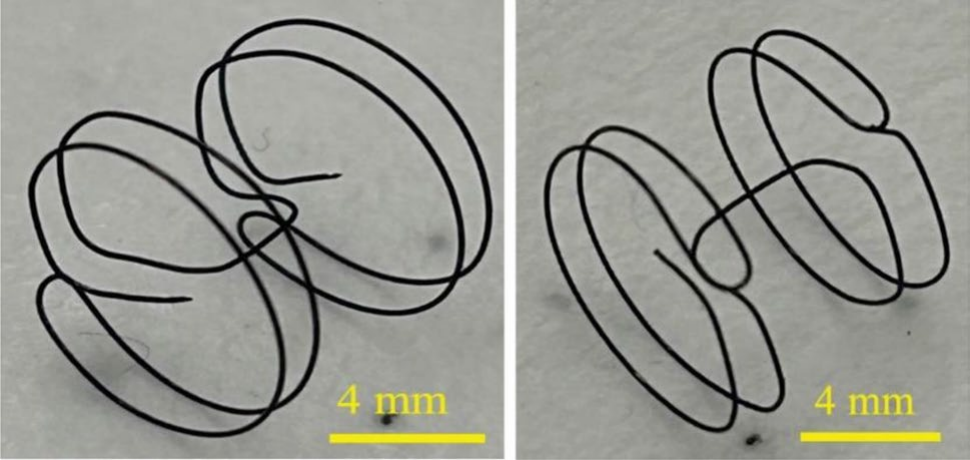
Nitinol with shape of Occluder. Enlarged view of NiTi frame (left and right are the same frame) with two pair of distinct concentric circular shaped walls connected at the center. This topology is suitable for occlusion of a defect on septum where it will locate at the center of the occluding disks.

**Figure 6:**
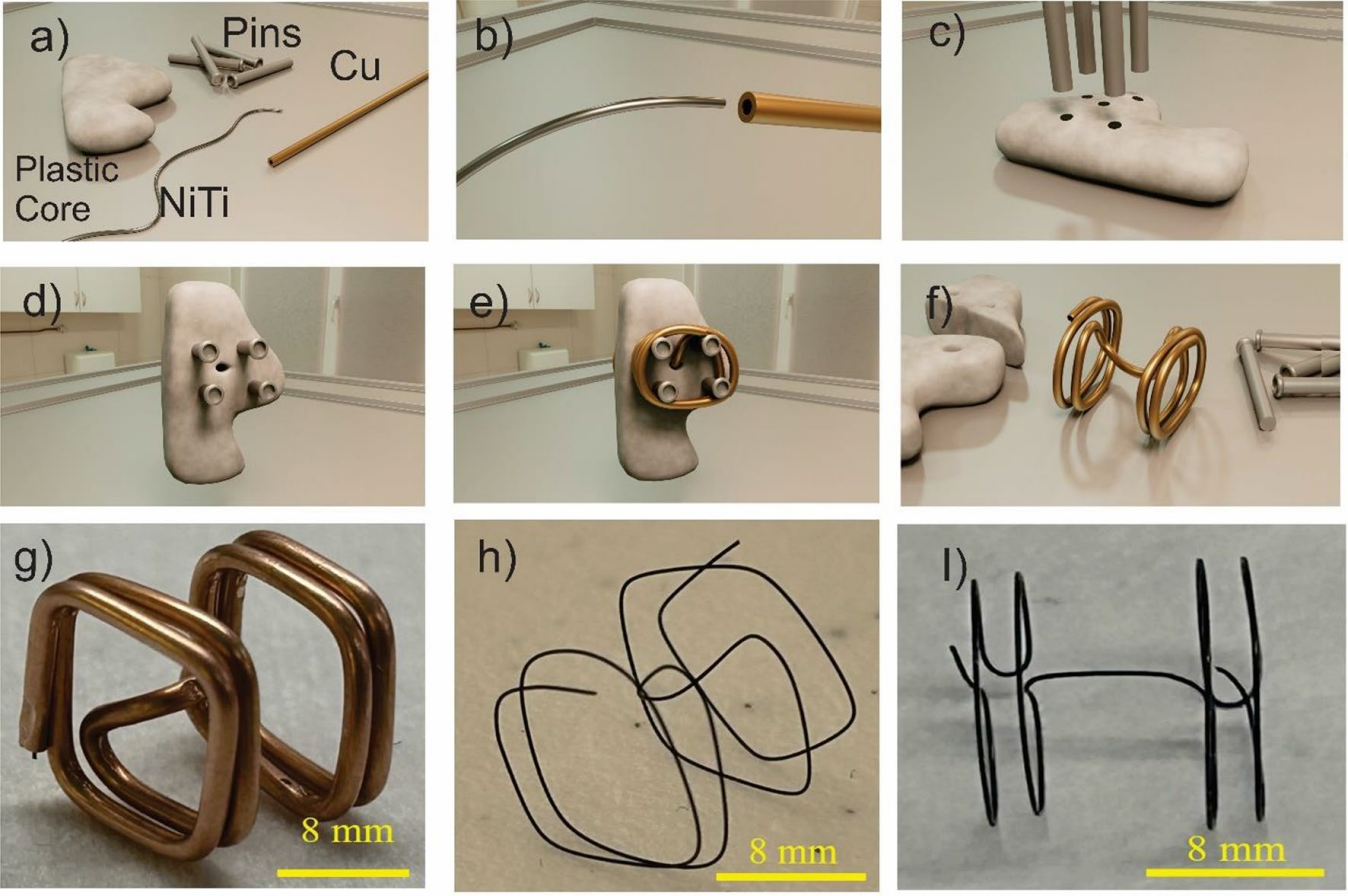
Use of a sacrificial polymeric cores and pins allows for starting with (**a**) superelastic NiTi wires and a core, (**b**) aligning NiTi inside copper, (**c**,**d**) aligning metal pins in a plastic core, (**e**) over which the Copper is wrapped, and (**f**) the composite of copper/NiTi with desired form be released after cutting the core to be annealed and etched, (**g**) occluder frame with shape of rectangles from experiment, (**h**) the occluder's frame after the heat treatment (**l**) and the frame after Etching.

We also fabricated other frames with the shape of septal defect occluder from 0.006″ diameter NiTi (Tegra Medical). To accomplish this, we first 3D printed an atrial septum, which served as the foundation for the septum fabrication (Fig. 3e). After 3D printing the septum (Fig. 3e), holes were marked and then drilled. Subsequently, bolts were threaded into the holes (Fig. 3f). Subsequently, we used this assembled structure as a core for winding NiTi wire reinforced by Cu (Fig. 3f). Once the NiTi winding was complete, we cut and removed the core (Fig. 6a–f). As a proof of concept, we fabricated occluders with different shapes, such as triangle (Fig. 3f) and rectangle (Fig. 6g–i). While there is no medical necessity for such rectangular/triangular shapes, we engineered these designs to showcase the versatility of the proposed technique. Additionally, we fabricated NiTi frames without any cores by manually bending wires using pliers (Fig. 3g, h). For example, we formed the nitinol/copper wire using handheld tools, following a trajectory of two connected disks (Fig. 3g). In another instance, we created NiTi wires that mimicked the shape of a septal occluder by bending the wire with pliers and mandrels (Fig. 3h). In this smaller frame (Fig. 3h), the disks were integrated with ChronoFlex® AR^44^, which is a hemocompatible^45,46^ polymer. Fabrication of the films involved spin coating ChronoFlex® AR, peeling off the cured films and bonding them together while sandwiching the NiTi disks (Fig. 3h). Another frame (Fig. 5) with the geometry suitable for anchoring on septum is fabricated without any cores in from 0.006″ in diameter NiTi wires.

Energy-dispersive X-ray spectroscopy (EDS) was performed on a NiTi wire (~ 0.003″) that was untreated and one (70 $\upmu$m diameter) that went through the proposed process. For this purpose, a S-3400N Type II Hitachi machine was used. The e-beam voltage was set at 20 kV. This voltage was associated with a penetration depth of ~ 1000 nm elsewhere^47^. Although the parameters in our work, such as materials used, may slightly affect this depth, we anticipate that the signal predominantly originates from the sample's surface. The EDS analysis recorded the counts for different voltages, and the obtained data is depicted in Fig. 4a and b. The analysis program allowed for the selection of various elements, with a focus on copper (Cu) in this particular analysis. Depending on the recorded signal, the program automatically included or excluded other elements from the analysis (Fig. 4a,b).

The dimensions of the structures in this study were measured with an optical microscope and a micrometer. For optical characterizations, multiple measurements were performed, and the average value along with the standard deviation was used to calculate the corresponding error bar with a 95 percent confidence level. This uncertainty is reported in Table 1 that is dominant to the expected uncertainty due to the diffraction limit of light (i.e., ~ ± 1 micron). The micrometers had an uncertainty of ± 1 micron. The calculation of the springs' pitch and diameters was carried out using an optical microscope in a similar manner. The statistical analysis excluded the first and last rings. To evaluate the repeatability of the process, nine additional springs were fabricated and characterized (see Table 1). In this process, other three springs were fabricated for practice and were not included in the statistical analysis. The springs in Figs. 1, 2a, and b were used to show the etch process and were not included in this analysis. The pitch and outer diameter of three samples from each spring type in Table 1 were optically characterized using a stereoscope (Amscope MU503). A small straight section of both untreated and heat-treated NiTi wire was also optically characterized to measure the wire diameter. Subsequently, the diameter of these two NiTi straight samples (heat-treated and untreated) was measured using a micrometer screw gauge to compare the measurements obtained from the optical characterization. The measured data underwent statistical analysis, primarily to calculate the average, standard deviation, and uncertainty. The error bars were determined using a Student's t-test.

**Table 1:** Three types of springs were fabricated using steel mandrels and their dimensions are characterized.

Mandrel type (needle)	Spring outside diameter (mm)	Intended diameter (mm)	Pitch (mm)	Wire diameter (microns)	No. tested
Gauge 27	0.67 ± 0.01	0.696	0.32 ± 0.02	72 ± 2	3
Gauge 21	1.09 ± 0.04	1.128	0.30 ± 0.01	73 ± 2	3
Gauge 16	1.87 ± 0.06	1.915	0.35 ± 0.02	73 ± 1	3

### Ethics declaration

The research presented did not use human or animal subjects. The patient’s image was taken from the publicly available^37,48,49^ resources at: https://viewer.imaging.datacommons.cancer.gov/viewer/1.2.826.0.1.3680043.10.474.6936989767597543453598603266931501661 with the Case ID: MIDRC-RICORD-1B-SITE2-000562. The image was reported as part of a dataset of de-identified CT scans through The Cancer Imaging Archive.

## Results

As shown in Fig. 1, stainless steel hypotubes (needles) could be used as a mandrel for winding 0.003″ diameter nitinol wires. Using this process, we observed the capability of forming a 0.003″ diameter wire in various structures including miniaturized springs with outer diameters of ~ 1.1 mm, ~ 0.7 mm, and ~ 2 mm (Fig. 2a,b). After the heat treatment, we observed black spots on the copper (Fig. 2a-2,2b-2) that could be attributed to copper III oxide. We observed that etching nitinol often required multiple steps, including dissolving crystalized copper sulfate in water, and etching again in fresh etchant solution. Figure 2b-3 shows an intermediate state of etching copper. As shown, copper is gradually etched without visible damage to the nitinol wire. After etching we optically characterized the nitinol springs (Fig. 2a-3,b-4) using an optical microscope. Finally, Scanning Electron Microscopy (SEM) shows an image of another spring formed by this process (Fig. 2c).

To further investigate the repeatability of the process, Table 1 presents the dimensions of 9 springs, all wound by a single person. It is noted that the change between the intended diameter and the actual diameter of the springs is limited to 5%. Similarly, the pitch uncertainty within each type of spring is restricted to 6%. An interesting observation is that the uncertainty in diameter increases from approximately 1.5–3% as the spring diameter increases. This indicates that larger-diameter springs exhibit a greater variation in dimensions.

The proposed method did not affect the dimensions of the NiTi wires or eliminate their plasticity. We conducted tests on the same wires before and after implementing the proposed process, and no changes were observed in diameter of the nitinol wires within the accuracy of our measurements (± 1 micron). Additionally, by subjecting the 0.003" diameter NiTi wire to heat treatment and etching processes, we confirmed a full recovery of at least 2% strain through simple bending of the wires over cylinders.

The tight variation of dimensions in the spring suggests the application of hypotubes for the fabrication of patient-specific self-expanding frames. To demonstrate this concept, the inter-atrial septum was segmented out from a de-identified individual’s CT images. Subsequently, silicone/PVA sacrificial cores with the shape of the septum are fabricated. Figure 3d shows the process of winding a wire around the septum. As shown the wire is plastically formed using pliers, then PDMS core is cut and removed. The wire was then heat treated and etched. We noted that as seen in Fig. 3d (inset-top) the shape of wire is not well smooth. This deficiency could be overcome by increasing the tension in the copper wire during the thermoplastic forming. This could be achieved by incorporating pins or mandrels on the segmented septum and then winding copper/NiTi composite wire. The result of this approach is shown in Fig. 3d (inset-bottom) where the shape of the wires is more round with minimal sharp edges.

The proposed approach demonstrated its capability for fabricating frames (0.006″ diameter wires) with irregular shapes, such as triangles (Fig. 3f) or cylindrical (Fig. 5), or rectangular sides (Fig. 6g–i). Furthermore, through wire bending techniques alone, it was possible to create a structure resembling two connected disks (Fig. 3g,h and Fig. 5) without the need for any cores. These structures can be integrated with hemocompatible polymers, offering a potential solution for blocking defects on the septum (Fig. 3h). This approach has great potential for fabricating patient-specific atrial septal occluders when combined with materials like Dacron or expanded Polytetrafluoroethylene (ePTFE).

EDS signals for NiTi wires (untreated and processed) are depicted in Fig. 4a,b. The software, while selecting Cu as a constituent, did not find its trace on the surface of NiTi. Here, as noted in the plot, a small portion of Al and Si was found (< 5% mass ratio) on the surface. Since these elements are not present in the chemical process, we attribute them to ambient contamination. In both samples about 10% carbon content was found which is typical for organic contaminations. We note that, due to the presence of organic and inorganic environmental contamination we cannot draw a conclusion based on the weight ratio of oxygen. Rather we rely on the weight ratio of Ti/Ni. The weight ratios of Ti and Ni on untreated and processed samples were both 45/55. Finally, the analysis was unable to find a trace of copper.

One can utilize sacrificial cores to facilitate the fabrication of complex topologies that are difficult to achieve using a traditional metal fixture. For example, a combination of sacrificial polymeric cores and metal pins/screws (illustrated in Fig. 6a–f) can be employed to wrap NiTi wires, resulting in rectangular shapes on both sides (Fig. 6g–i) that was fabricated in this work. This technique enables the creation of intricate self-expanding frames such as those with rectangular shapes, as demonstrated in Fig. 6g–i.

## Discussions

The variation in the fabricated springs (~ 3% in diameter; Table 1) raises the question of what may limit the accuracy of the intended dimension in this process. Moreover, our observation suggested that the larger-diameter spring may show a larger variation. One of the mechanisms that may limit this accuracy originates from the strain stored in the wire while it is being deformed. In the proposed process, the core/template is removed after formation of the desired structure and before the heat treatment, in contrast with the traditional use of fixtures. This allows a small variation of the structure’s dimensions due to the elastic pre-strain in copper or nitinol. If we ignore the pre-strain in the nitinol wire, we can state that elastic strain stored in the copper hypotube may result in the change of the tube’s radius of curvature, after removing the core, as follows:

1 $$\epsilon_{Cu - Elastic} = - d\frac{{{\Delta }R}}{{2R^{2} }}$$

where ‘*d’* is the diameter of the Cu hypotube, ‘*R’* is the radius of curvature for the copper/NiTi wires and ‘${\Delta }R$’ is the change in the radius of curvature before and after releasing the elastic strain stored in Cu. Since $\epsilon_{Cu - Elastic}$ is the material property, then we can expect the percentage variation in the radius of curvature to be: $\frac{{{\Delta }R}}{R} = 2\epsilon_{Cu - Elastic} R/d$. Assuming the worst-case scenario for $\epsilon_{Cu - Elastic}$ to be the yield strain (~ 0.002) ^50^, and $R/d$ to be ~ 6 (e.g., the springs in Table 1 with OD ~ 1.9 mm) we calculate the variation to be ~ 2%. This variation is in the same order of magnitude consistent with what we observed in terms of the variation of the dimensions for the springs. Furthermore, using Eq. (1), we estimate that for a ring with a diameter of 5 mm, a variation of ~ 5% change in the diameter is expected. This concept allows us to provide a tool to predict the variation of dimensions after releasing NiTi/Cu structures from the sacrificial core.

Another observation was that the control over the variation of the radii of curvature for each location of the copper is challenging, as seen in Fig. 3d, where radius of curvature is significantly larger than in the springs (Fig. 2). This can be understood in view of the fact that for an elastoplastic forming process, the strain must exceed the yield limit in Eq. 1. However, when *R* is large in $\frac{{d{\Delta }R}}{{2R^{2} }}$ inducing a large strain by pure bending might be insufficient to achieve a strain beyond the yield strain in copper. The consequence of this issue can be seen in Fig. 3d-inset-top. Here only pure bending was used (using pliers), and therefore the control over strain in the structure was challenging. Therefore, this notion offers an explanation for why the dimensions of the copper wire are highly uneven for this structure as opposed to the miniaturized springs in Fig, 2. This issue can simply be improved by either (1) adding tension in the wire during the forming process and therefore controlling the curvature more efficiently or (2) using wires with larger diameter *d* (Fig. 5). In Fig. 3d-inset-bottom, nitinol/copper wires were winded around a pin that was passed through the patient-specific implant. This allowed forming of the copper wire with high tension, and therefore it retained the circular shapes on the two disks more evenly.

Further demonstration of the versatility of the proposed technique, NiTi frames with larger diameters (Fig. 5) exhibit enhanced control over dimensions when utilizing thicker wire. Notably, these frames (Fig. 5) were fabricated solely through wire bending without the need for any cores. Furthermore, the bar connecting the two disks are located at the center of the disks (as to compared with 3g or 3d), which is suitable for the design of a typical septal defect occluder. This process also highlights the potential for utilizing computerized wire bending machines for rapid prototyping of complex topologies. Additionally, the versatility of this method is demonstrated by the fabrication of NiTi super-elastic frames with triangular shapes (Fig. 3f) and rectangular plugs (Fig. 6g). By employing a combination of polymeric cores and pins (as shown in Fig. 6), a NiTi frame that follows the anatomy of the atrial septum is successfully created. Furthermore, to demonstrate the capability of this method, plugs with triangular shapes (Fig. 3f) as well as rectangles (Fig. 6g–i) are formed. Although these shapes may not possess direct medical advantages, they demonstrate the utility of the technique when irregular shapes are required.

The EDS analysis of the 0.003″ NiTi wires (Fig. 4a,b) revealed a consistent weight ratio of Ti and Ni on the wire surface within a depth of approximately 1 micron, with a ratio of 45/50. This weight ratio aligns with the typical 50% molar ratio for nitinol. Importantly, this ratio remained unchanged from the unprocessed NiTi wire in comparison to the wire that underwent annealing and etching processes. This finding strengthens our confidence that the proposed annealing and etching processes have a minimal impact on the elemental ratio of nitinol at the micro-scale. Moreover, the absence of a copper (Cu) signature in the EDS analysis suggests that the presence of Cu is insignificant at the micro-scale. We note that the EDS analysis result is preliminary at this stage, which does not show a large amount of copper (Cu). It is still possible that any undesirable phases, such as Ni-rich or Cu-rich regions, if present, exist at sub-micron depths. However, further quantitative investigations using more sensitive tools are warranted to study the processed NiTi and identify any potential undesirable phases. In the event that such phases are found, traditional etching processes can be employed to remove them without significantly affecting the dimensions of the NiTi structure, given their expected location on the surface of the wires.

The possibility of individualized implants has attracted researchers’ attention to the advancement in rapid prototyping techniques^8,28, 44, 51^. Nitinol is among the most critical materials for implantations. While additive manufacturing introduced technologies for rapid fabrication of implants made from hemocompatible/biocompatible materials such as Tecoflex^TM^, ChronoFlex®, PDMS and Titanium, the progress in a low-cost and fast method for shape setting nitinol has been limited. Direct 3D printing of nitinol is challenging and fabrication of metal fixtures is expensive. Here, the proposed technique introduces an addition to the previous technologies that will enable future work on individualized implants. While we envision this technique to be adaptable to a wide range of medical applications here, as a proof of concept, we demonstrate its capability for the rapid prototyping of an individualized atrial occluder (Fig. 5). We note that we did not define the need for a patient-specific implant based on the medical images, rather we provide a demonstration of the versatility of proposed technique with potentials for fabrication of patient-specific implants with complex shapes.

The introduced process offers improvements in rapid prototyping of NiTi structures that leverage additive manufacturing, and digital manufacturing. First, it enables the use of low-cost 3D-printed polymers as the cores or fixtures for shape setting nitinol. Although direct 3D printing NiTi^52^, metal fixtures^7^, or casting ceramic fixtures^8^ have been demonstrated, this work demonstrates the use of low-cost FDM 3D printed parts instead of metal/ceramic fixtures. This use of polymeric 3D printed fixtures offers a significant reduction in the cost or turnaround time as compared with 3D printing metal fixtures. Another promising possibility is an integration of Cu/NiTi wires with, desktop-scale computerized wire bending machines^39^ that are available for rapid prototyping^53^. This approach can eliminate the need of fixtures in certain topologies and manual operations, which reduces the turnaround time for fabrication of frames made from crimped nitinol wires (e.g., heart valves^2,33^ in medical implants, and superplastic mesh tires^30,31^ used in the aerospace industry). Finally, we envision that Cu/NiTi can be integrated with other additive manufacturing techniques that process continuous fibers such as automated fiber placement^54^ or continuous fiber 3D printers^55^ for the fabrication of complex topologies of NiTi/Cu topologies. This technology, if integrated with automated crimping tools, can offer exciting opportunities for the rapid prototyping of complex NiTi structures.

Future work is envisioned to explore the limits of the proposed fabrication method and its potential. Use of NiTi in medical implants requires significant preclinical studies that are envisioned to be addressed in the future^56–58^. Specifically, the effects of processes such as etching on the long-term durability and biocompatibility of the frames should be investigated. Further studies on the surface chemistry and biocompatibility of NiTi frames may also reveal the need for additional processes such as etching NiTi or the use of vacuum ovens or salt baths. Another important process parameter to study is the nitinol pre-strain, which is known to impact its fatigue life. Exploring the integration of the proposed technique into computerized wire bending machines or continuous fiber 3D printers could demonstrate novel methods for rapid prototyping of NiTi. Considering that many implants' frames are fabricated in the form of sheets and tubes, introducing composite NiTi sheets and tubes reinforced with copper may significantly expand the utility of the proposed technique in the future. Such reinforcement can be achieved by aligning NiTi sheets/tubes, that are patterned by laser cutters, between two Cu tubes/sheets. Subsequently the outer and inner Cu layers can be spot welded through perforated NiTi areas. This process can produce composite layers of Cu/NiTi/Cu, in the forms of sheets or tubes. Subsequently such structures can be formed by cold forging or cold bending processes. Future work is also envisioned to integrate rapid prototyping of NiTi with other hemocompatible materials (such as Dacron or ePTFE) to facilitate the fabrication of personalized implants.

In summary, we proposed a technique for fabrication of complex NiTi frames from nitinol wires reinforced by sacrificial tubes or hypotubes that were made from copper. In this fashion, upon the bending of nitinol wires, the sacrificial tube deforms (with plastic deformation) at room temperature and creates a permanent supporting fixture. We showed that the sacrificial cores could be removed and the copper hypotubes can serve as a fixture for the heat treatment of nitinol. We demonstrated that miniaturized springs can be fabricated using this method. It was demonstrated that this technique is capable of the rapid prototyping self-expanding frames with possible applications in medical technologies. For a demonstration of the concept, we fabricated NiTi frames with a topology for anchoring on a atrial septum. We further showed that those frames could be customized with the shape of the septum or irregularly shaped plugs. We propose a metric to estimate the control over the dimensions and show that this technique is ideal for the rapid prototyping of NiTi frames with smaller radii of curvature. The capability of this technique in producing complex topologies suggests that this technique have the potential to be integrated with computerized wire forming techniques. These results have significant applications in the rapid manufacturing of nitinol structures applicable in various areas, such as patient-specific or individualized implants.

## Data Availability

The dicom files used for segmentation is available through: https://portal.imaging.datacommons.cancer.gov/explore/filters/?collection_id=midrc_ricord_1b (Case ID: MIDRC-RICORD-1B-SITE2-000562). 3d Slicer software is publicly available through: https://www.slicer.org. Other data necessary to replicate the findings are available upon request from the corresponding author.
